# Lesion‐to‐anal‐verge distance in rectosigmoid endometriosis on transvaginal sonography *vs* magnetic resonance imaging: prospective study

**DOI:** 10.1002/uog.26083

**Published:** 2023-02-01

**Authors:** M. K. Aas‐Eng, V. S. Young, J. B. Dormagen, A. H. Pripp, G. Hudelist, M. Lieng

**Affiliations:** ^1^ Department of Gynecology Oslo University Hospital Oslo Norway; ^2^ Institute of Clinical Medicine, Faculty of Medicine University of Oslo Oslo Norway; ^3^ Department of Radiology and Nuclear Medicine Oslo University Hospital Oslo Norway; ^4^ Oslo Center for Biostatistics and Epidemiology Oslo University Hospital Oslo Norway; ^5^ Department of Gynecology, Certified Center for Endometriosis and Pelvic Pain Hospital St John of God Vienna Austria; ^6^ Rudolfinerhaus Private Clinic Vienna Austria; ^7^ Stiftung Endometrioseforschung/Endometriosis Research Group DACH Region, Central Europe; ^8^ Division of Obstetrics and Gynecology Oslo University Hospital Oslo Norway

**Keywords:** bowel endometriosis, complication risk, deep‐infiltrating endometriosis, magnetic resonance imaging, MRI, rectosigmoid endometriosis, surgical planning, surgical treatment, transvaginal sonography

## Abstract

**Objectives:**

To compare transvaginal sonography (TVS) and magnetic resonance imaging (MRI) with intraoperative measurement (IOM) using a rectal probe in the estimation of the location of rectosigmoid endometriotic lesions, i.e. lesion‐to‐anal‐verge distance (LAVD), and to compare two different MRI techniques for measuring LAVD.

**Methods:**

This was a prospective single‐center observational study that included women undergoing surgery for symptomatic rectosigmoid endometriosis by discoid (DR) or segmental (SR) resection from December 2018 to December 2019. TVS and MRI were performed presurgically for each participant to evaluate LAVD, and the measurements on imaging were compared with IOM using a rectal probe. Clinically acceptable difference and limits of agreement (LoA) between TVS and MRI compared with IOM were set at ± 20 mm. Two different measuring methods for MRI, MRI_Center_ and MRI_Direct_, were proposed and evaluated, as there is currently no guideline to describe deep endometriosis on MRI. Bland–Altman plots and LoA were used to assess agreement of TVS and both MRI methods with IOM. Systematic and proportional biases were assessed using paired *t*‐test and Bland–Altman plots.

**Results:**

Seventy‐five women were eligible for inclusion. Twenty‐eight women were excluded, leaving 47 women for the analysis. Twenty‐three DR and 26 SR procedures were performed, with both procedures performed in two women. The Bland–Altman plots showed that there were no systematic differences between TVS or MRI_Center_ when compared with IOM for all included participants. MRI_Direct_ systematically underestimated LAVD for lesions located further from the anal verge. TVS, MRI_Center_ and MRI_Direct_ had LoA outside the preset clinically acceptable difference when compared with IOM. LAVD was within the clinically acceptable difference from IOM of ± 20 mm in 70% (33/47) of women on TVS, 72% (34/47) of women on MRI_Center_ and 47% (22/47) of women on MRI_Direct_.

**Conclusions:**

TVS should be the preferred method to estimate the location of a rectosigmoid endometriotic lesion, i.e. LAVD, as it is more available, less expensive and has a similar accuracy to that of MRI. Estimating LAVD can be relevant for planning colorectal surgery for rectosigmoid endometriosis. © 2022 The Authors. *Ultrasound in Obstetrics & Gynecology* published by John Wiley & Sons Ltd on behalf of International Society of Ultrasound in Obstetrics and Gynecology.

**CONTRIBUTION**

*What are the novel findings of this work?*

Transvaginal sonography (TVS) and magnetic resonance imaging (MRI) have a similar performance in the assessment of location of rectosigmoid endometriotic lesions.

*What are the clinical implications of this work?*

Detailed mapping of the extent of deep endometriosis, including determining the location of a rectosigmoid endometriotic lesion using TVS or MRI, provides information that may improve counseling of women and assessment of risk of complications to optimize surgical planning.

## INTRODUCTION

Deep endometriosis (DE) infiltrating the rectosigmoid is a benign condition that can cause symptoms including cyclical or chronic pelvic pain, painful defecation, altered bowel function and dyspareunia^1^. Endometriosis can have substantial personal and socioeconomic impact, and DE is associated with increased pain severity^1^. Diagnostic delay may hinder treatment^2^. If medical treatment of rectosigmoid endometriosis fails, surgical treatment with shaving, discoid resection (DR) or segmental resection (SR) of the bowel may be necessary^3^, ^4^. Potential serious surgical complications and risk of need for a temporary stoma pose a challenge for women and the gynecological surgeon. Thus, it is pertinent to record the extent of DE^5^, ^6^ with a systematic description as proposed by the International Deep Endometriosis Analysis (IDEA) group^7^. A recent study has shown that the risk of rectovaginal fistula increases when the rectosigmoid endometriotic lesion is ≤ 80 mm from the anal verge^8^. Data from rectal cancer surgery demonstrate that an anastomotic height of < 50 mm from the anal verge is associated with a 4‐fold increase in anastomotic leakage^9^. Therefore, to assess the risk of anastomotic leakage and need for a temporary stoma, it is vital to measure the location of the rectosigmoid endometriotic lesion with respect to the anal verge, hereafter termed the lesion‐to‐anal‐verge distance (LAVD). Our group has shown recently that transvaginal sonography (TVS) is a valuable tool for estimation of LAVD presurgically to estimate the final height of the anastomotic stapling lines^10^. TVS and magnetic resonance imaging (MRI) are established tools for diagnosing DE; however, MRI may not be as readily available as TVS. In contrast to TVS, there is no guideline describing how to report DE findings on MRI. It is unknown how well TVS performs compared with MRI in measuring LAVD in rectosigmoid endometriosis.

The aim of the present work was to evaluate the performance of MRI and TVS, based on the IDEA‐group guidelines^7^, compared with intraoperative measurement (IOM) for assessment of LAVD. We also aimed to compare the performance of two different MRI techniques for measuring LAVD, namely MRI_Center_ and MRI_Direct_.

## METHODS

This was a prospective single‐center observational study of women scheduled for elective surgery with DR or SR due to symptomatic rectosigmoid endometriosis who were recruited consecutively from a tertiary referral center for endometriosis at Oslo University Hospital, Oslo, Norway, between December 2018 and December 2019. The participants were included at the time of recruitment for two other multicenter studies, but the other centers did not have MRI data available for the current study^10^, ^11^. Women who underwent shaving were excluded, as they did not undergo full‐thickness resection of the bowel with a clear surgical anastomosis. Exclusion criteria were previous bowel surgery, age < 18 years, virginity and menopause^10^. All women included in the study had undergone TVS and MRI before surgical management with DR, SR or both for rectosigmoid endometriosis. The clinically acceptable difference between the measurement on TVS or MRI and the reference standard IOM was established as ± 20 mm. Thus, if the IOM was 100 mm and LAVD was underestimated at 80 mm by TVS or MRI, the estimated LAVD would still indicate a low risk of need for a temporary stoma, anastomotic leakage and rectovaginal fistula^8^. The study was approved by the regional ethics committee for medical research in Norway (reference REK 2017/1925).

### Transvaginal sonography

**Figure 1:**
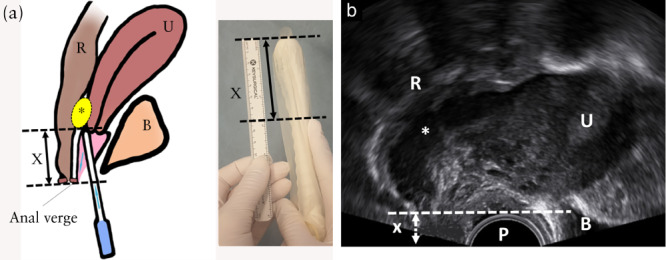
Method 1 for measuring lesion‐to‐anal‐verge distance (LAVD) of a rectosigmoid endometriotic lesion (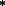) located at/near the rectovaginal septum, using transvaginal sonography (TVS), demonstrated schematically (a) and on TVS (b). The inferior part of the lesion is identified and the tip of the TVS probe is placed at this level. The anal verge is visualized, and the index finger is placed on the TVS probe at the level of the anal verge. LAVD is measured with a ruler on the TVS probe from the distal tip of the probe to the index finger. B, bladder; P, TVS probe; R, rectosigmoid; U, uterus; X, LAVD.

**Figure 2:**
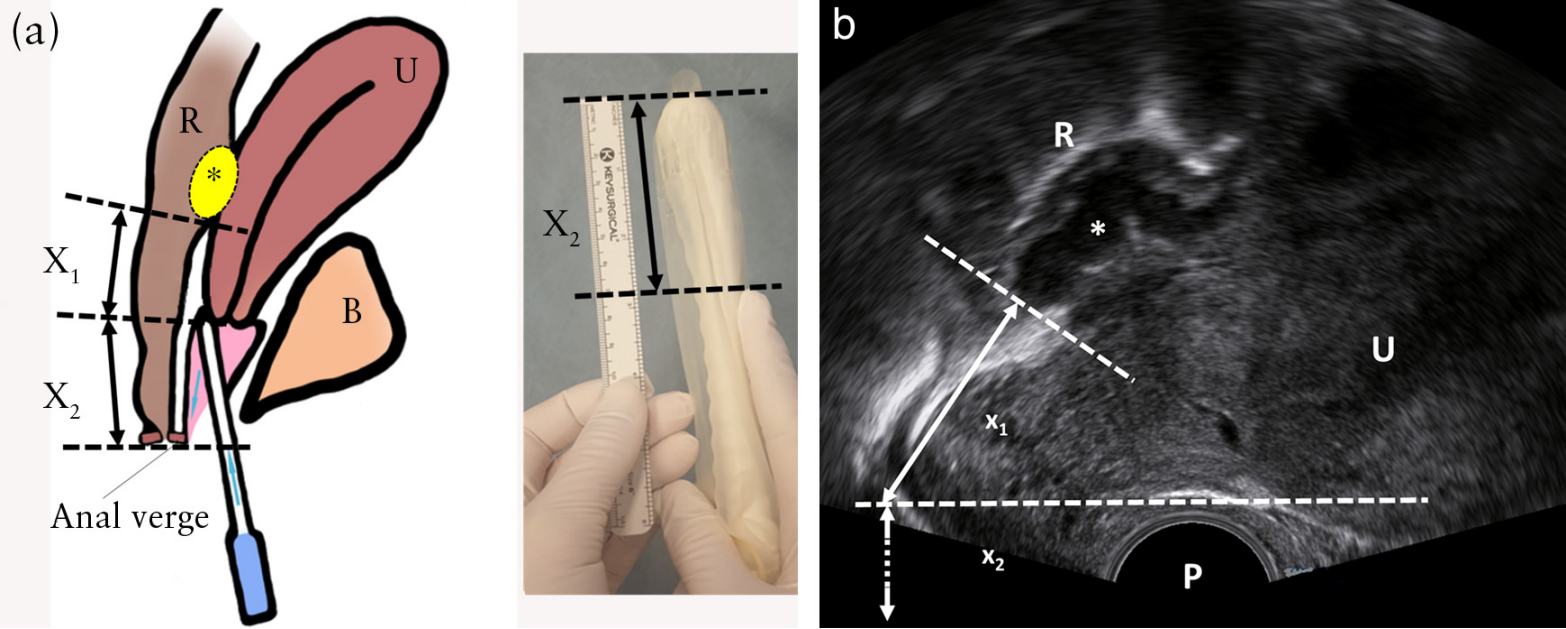
Method 2 for measuring lesion‐to‐anal‐verge distance (LAVD) of a rectosigmoid endometriotic lesion (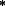) located cranially to the rectovaginal septum, using transvaginal sonography (TVS), demonstrated schematically (a) and on TVS (b). The inferior part of the lesion and the lower lip of the posterior cervix are identified on TVS in a frozen image, and the distance between is measured (X_1_). The tip of probe is placed at the lower lip of the posterior cervix. The anal verge is visualized, and the index finger is placed on the vaginal probe at this level (X_2_). X_2_ is measured with a ruler on the TVS probe from the distal tip to the index finger. Total LAVD is calculated by adding X_1_ and X_2_. B, bladder; P, TVS probe; R, rectosigmoid; U, uterus.

Ultrasound examination was performed by one examiner (M.K.A.) who had 7 years' experience with TVS in gynecology and was blinded to MRI measurements. The examination was performed using a 5–9‐MHz transvaginal probe with three‐dimensional (3D) facility (WS80A; Samsung Healthcare, Seoul, South Korea). The anterior and posterior compartments of the pelvis were examined systematically using TVS^7^. On TVS, a rectosigmoid endometriotic lesion appears as a hypoechoic thickening or nodule, usually affecting the muscularis propria of the bowel wall^7^. LAVD was measured by two different methods depending on the level of the lesion with respect to the rectovaginal septum (RVS), as illustrated previously^10^, based on the recommendations of the IDEA group^7^. Method 1: for lesions below or at the level of the RVS, the tip of the probe was set at the caudal part of the lesion and an index finger was placed on the TVS probe at the visualized and estimated level of the anal verge. The probe was withdrawn and the distance from the tip of the probe to the index finger was measured using a ruler, representing the LAVD (Figure 1). Method 2: for lesions above the level of the RVS, the distance from the caudal part of the lesion to the posterior cervix was measured in a frozen image (LAVD‐1), and the distance from the lower lip of the posterior cervix to the anal verge (LAVD‐2) was measured using Method 1. The sum of LAVD‐1 and LAVD‐2 represented the total LAVD (Figure 2). If the woman had undergone total hysterectomy or supracervical hysterectomy, Method 1 was used for lesions below or at the level of the vaginal cuff or cervix and Method 2 was used for lesions above that level. Total LAVD consisted of the distance from the caudal part of the lesion to the posterior part of the vaginal cuff or the posterior lip of the cervix in a frozen image (LAVD‐1) and the distance from the vaginal cuff or lower lip of the posterior cervix to the anal verge measured using Method 1 (LAVD‐2). The most caudal rectosigmoid endometriotic lesion closest to the anal verge was measured, as this would be the site for rectosigmoid anastomosis^10^. No bowel preparation was utilized, as this is not routine practice in our department.

### MRI sequences

At Oslo University Hospital, MRI sequences for the diagnosis of endometriosis are performed in line with the European Society of Urogenital Radiology guidelines^12^. MRI examination was performed using a 1.5‐Tesla (T) Philips Achieva (Philips Medical Systems, Best, The Netherlands) with a 32‐channel cardiac coil and 1.5‐T Siemens Aera (Siemens Healthineers, Erlangen, Germany) with a 30‐channel body coil. A 3D T2‐weighted (T2W) balanced turbo field echo was acquired in the axial plane with sagittal and coronal reformates from the promontory to below the anal verge. At the location of DE infiltrating the rectosigmoid, one high‐resolution two‐dimensional (2D) T2W turbo spin echo was performed perpendicular to the rectosigmoid involvement, with the angulation controlled by a radiologist. Axial T1‐weighted (T1W) 2D Dixon technique generated four simultaneous T1W sequences, including a sequence with fat suppression, which is a crucial technique for detecting blood foci. Prior to MRI, women fasted for 4 h, were administered a rectal suspension (Toilax Micro Enema; Orion Corp., Orion Pharma, Kuopio, Finland (10 mg/5 mL)), performed voiding of the bladder and received 20 mg of butylscopolamine (Buscopan®; Sanofi‐Aventis, Reading, UK) intravenously and 1 mg of glucagon intramuscularly. All MRI images were stored anonymously on the Syngo Imaging picture archiving and communication system (Siemens Healthineers). The radiologist (V.S.Y.) had over 16 years' experience with endometriosis and abdominal MRI and was blinded to the sonographic data. In 14 cases, MRI had been performed at another institution, and the acquired images were retrieved and reassessed. MRI sequences in 13 of these cases from the other hospitals were similar to the method described above, although some minor differences may have occurred, which did not affect image interpretation. In one case, MRI was performed at a private institute that did not have the same MRI sequence as the other hospitals. The image in that case was assessed to be of acceptable quality and was thus included.

#### MRI interpretation

**Figure 3:**
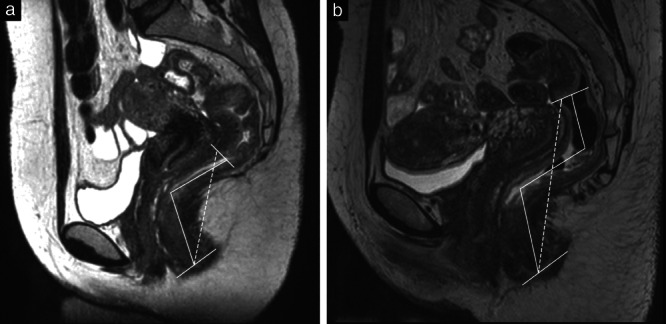
Magnetic resonance images (MRI) in two patients with rectosigmoid endometriosis, showing measurements of lesion‐to‐anal‐verge distance using MRI_Direct_ (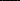) and MRI_Center_ (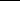) methods.

The typical appearance of endometriotic lesions in the bowel is a fan‐shaped configuration in the anterior part of the rectal wall, located in the upper part of the rectum or the rectosigmoid. Bowel wall endometriosis has low signal intensity or isointense signal (compared with the muscle) on T2W and T1W images, depending on the fibrotic reaction. Moreover, small hemorrhagic foci can be detected easily on the fat‐suppressed T1W images. There is currently no guideline describing how to report DE findings on MRI, similar to the IDEA consensus for ultrasound. The anal verge as an anatomical landmark is not visible on MRI, only on clinical examination^13^. Research on MRI in rectal cancer has shown that there is up to 55‐mm variation in tumor height depending on the landmark used^14^. In this study, we used the intersphincteric groove, which has been suggested by some studies as a suitable landmark when describing rectal tumors on MRI^15^. Adapted from the studies by Han *et al*.^16^ and Bates *et al*.^15^, we employed two methods for measuring LAVD on MRI. The MRI_Direct_ method involved one straight‐line caliper from the midline of the intersphincteric groove to the most caudal and anterior part of the rectosigmoid endometriotic lesion (Figure 3). The MRI_Center_ method involved more than one straight‐line caliper from the midline of the intersphincteric groove, drawn in the center of the bowel lumen, to the most caudal part of the rectosigmoid endometriotic lesion (Figure 3). We hypothesized that more caudal lesions located closer to the anal verge with one caliper line (MRI_Direct_) would reflect better the straight rectal probe used for IOM compared with more cranial lesions located further from the anal verge using several calipers (MRI_Center_). The clinically acceptable difference between MRI and IOM was set as ± 20 mm.

### Surgical procedure and IOM

The surgical procedure was performed by a multidisciplinary DE team including gynecologists, general surgeons and urologists^4^, ^10^. After surgical dissection and detachment of the rectosigmoid endometriotic lesion from the surrounding structures, a rectal probe was inserted up to the caudal infiltration margin in a tension‐free state, which was considered as the reference standard. The caudal part of the lesion was visualized and palpated intraoperatively and the anal verge was marked with the index finger on the probe^10^. The rectal probe was removed and a ruler was used to measure the LAVD. All measurements were made by the same examiner (M.K.A.) who was blinded to TVS and MRI measurements at the time of IOM. All colorectal anastomoses ≤ 25 cm from the anal verge were inspected and air‐leak tested using saline in the pelvic cavity and insufflation of the rectosigmoid using a rectoscope. The surgical procedure for DR and SR has been described in detail previously^4^. The severity of disease was classified according to the revised American Society for Reproductive Medicine (rASRM) score^17^ and Enzian score^18^.

### Statistical analysis

Bland–Altman plots and LoA^19^, ^20^ were used to examine the agreement in LAVD measurements between TVS *vs* IOM and MRI_Center_ or MRI_Direct_ *vs* IOM. The Bland–Altman plots illustrate the differences between two measuring methods against the mean of the two measurements for each woman. They allow detection of any systematic differences between the methods. The upper and lower LoA are defined as the mean difference between the measuring methods ± 1.96 SD of the mean difference. LoA are the interval within which 95% of the differences between the measuring methods would lie if the study was repeated. LoA represent the range of differences in scores between the measuring methods (TVS and IOM or MRI and IOM) for other similar subjects measured under similar conditions. Before the study was undertaken, clinically acceptable LoA were set at ± 20 mm. Hence, 95% of differences in LAVD between the measuring methods, TVS or MRI compared with IOM, should lie between these limits if the study was repeated. Average differences in LAVD measurements between TVS, MRI_Center_ or MRI_Direct_ *vs* IOM were tested for statistical significance using the paired sample *t*‐test, and the significance level was set at 0.05. No power calculation was performed, as the aim of the study was to observe and describe any differences in LAVD between two established diagnostic tools. The analysis was performed using IBM SPSS Statistics for Windows, version 27.0 (IBM Corp., Armonk, NY, USA).

## RESULTS

**Figure 4:**
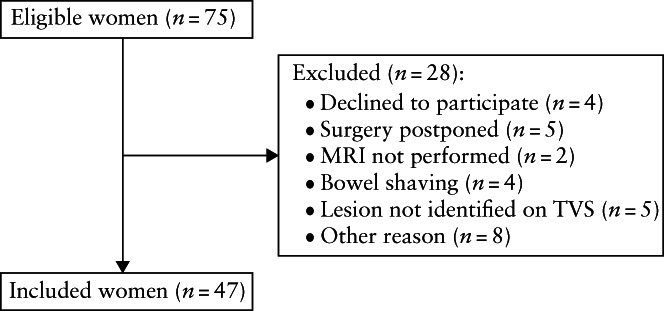
Flowchart summarizing inclusion of women scheduled for surgery for rectosigmoid endometriosis undergoing presurgical magnetic resonance imaging (MRI) and transvaginal sonographic (TVS) measurement of lesion‐to‐anal‐verge distance.

Seventy‐five women from Oslo University Hospital, Oslo, Norway were eligible for inclusion. Twenty‐eight women were excluded, leaving 47 women for analysis (Figure 4). Demographic details, surgical procedures and anastomotic height are presented in Table 1. Due to an anastomotic height of ≤ 70 mm, two women underwent temporary ileostomy at the time of primary surgery. Table 2 presents LAVD measurements and data on anatomical location of rectosigmoid endometriotic lesions^21^ obtained using the different methods. Systematic bias and LoA for the LAVD measurements obtained using the different methods are summarized in Table 3.

**Table 1:** Demographic and medical characteristics of 47 women with rectosigmoid endometriosis scheduled for surgery

Characteristic	Value
Age (years)	38.3 ± 6.2
BMI (kg/m^2^)	24.9 ± 4.8
Symptoms	
Dysmenorrhea	44 (93.6)
Dyschezia	46 (97.9)
Dyspareunia	36 (76.6)
Constipation	33 (70.2)
Diarrhea	27 (57.4)
Dysuria	16 (34.0)
Rectal bleeding	13 (27.7)
Infertility	15 (31.9)
Previous surgery for endometriosis	
0	9 (19.1)
1	25 (53.2)
> 1	13 (27.7)
rASRM score at surgery	
I	0 (0)
II	6 (12.8)
III	21 (44.7)
IV	20 (42.6)
Enzian classification of DE on TVS	
C1 (rectosigmoid lesions < 1 cm)	0 (0)
C2 (rectosigmoid lesions 1–3 cm)	26 (55.3)
C3 (rectosigmoid lesions > 3 cm)	21 (44.7)
Surgical technique	
Discoid resection*	23 (48.9)
Segmental resection*	26 (55.3)
Preoperatively planned ileostomy	2 (4.3)
Emergency stoma	0 (0)
Surgical anastomotic height† (mm)	106.9 ± 26.0

Data are given as mean ± SD or *n* (%).

* Discoid resection and segmental resection were performed during the same surgery in two women.

† Measured with rectoscope by general surgeon.

(Data missing for two women.) BMI, body mass index; DE, deep endometriosis; rASRM, revised American Society for Reproductive Medicine; TVS, transvaginal sonography.

**Table 2:** Lesion‐to‐anal‐verge distance (LAVD) and anatomical site of rectosigmoid endometriotic lesion, according to assessment by intraoperative measurement (IOM), transvaginal sonography (TVS) or two different methods of magnetic resonance imaging (MRI_Center_ and MRI_Direct_)

	IOM	TVS	MRI_Center_	MRI_Direct_
Characteristic	(*n* = 47)	(*n* = 47)	(*n* = 47)	(*n* = 47)
LAVD (mm)	118.6 ± 33.7	116.9 ± 21.2	127.0 ± 34.9	91.2 ± 14.6
	120.0 (60.0–230.0)	114.2 (59.4–167.7)	118.2 (66.7–267.7)	89.9 (58.5–119.7)
Anatomical site^21^				
Lower rectum (0–5 cm)	0	0	0	0
Midrectum (> 5–10 cm)	16	8	9	34
Upper rectum (> 10–15 cm)	25	35	31	13
Sigmoid (> 15 cm)	6	4	7	0

Data are given as mean ± SD, median (range) or *n*.

**Table 3:** Agreement in measurement of lesion‐to‐anal‐verge distance by magnetic resonance imaging techniques (MRI_Center_ and MRI_Direct_) or transvaginal sonography (TVS) *vs* intraoperative measurement (IOM) in 47 women with rectosigmoid endometriosis

	Mean difference (95% CI)		Limits of agreement
Comparison	(mm)	*P*	(mm)*
MRI_Center_ *vs* IOM	8 (3 to 14)	0.006	48 to −31
MRI_Direct_ *vs* IOM	−27 (−35 to −19)	0.001	26 to −81
TVS *vs* IOM	−2 (−10 to 6)	0.67	50 to −54

* If study was repeated, 95% of differences between measuring methods would lie between these limits.

All measuring methods had LoA outside the *a‐priori* set LAVD difference of ± 20 mm. LoA demonstrate that TVS, MRI_Center_ and MRI_Direct_ would not be able to measure LAVD within ± 20 mm of IOM in 95% of other subjects measured under similar conditions. Differences in LAVD may be expected to be up to ± 48 mm between MRI_Center_ and IOM and up to ± 54 mm between TVS and IOM (Table 3 and Figure S1a,b). Differences in LAVD on MRI_Direct_ compared with IOM could be up to ± 81 mm (Table 3 and Figure S1c).

There was no tendency for MRI_Center_ and TVS to systematically under‐ or overestimate LAVD compared with IOM (Table 3 and Figure S1a,b). On the other hand, MRI_Direct_ tended to underestimate LAVD compared with IOM for rectosigmoid DE lesions located further from the anal verge (Table 3 and Figure S1c). Removal of the outlier in the Bland–Altman plots with the greatest difference between the measuring methods for MRI_Direct_ and TVS (Figure S1c) did not affect the agreement analysis. The same participant was not an outlier on MRI_Center_, indicating that MRI_Center_ may be better at measuring LAVD of lesions located further from the anal verge. LAVD measurement was lower on TVS in 23 (48.9%) and higher in 22 (46.8%) women compared with IOM. There was perfect agreement in LAVD between TVS and IOM in two (4.3%) women. LAVD measurement on MRI_Center_ was lower in 12 (25.5%) and higher in 35 (74.5%) women compared with IOM. LAVD on MRI_Direct_ measured lower in 46 (97.9%) and higher in one (2.1%) woman. MRI_Center_ and TVS had good precision in measuring LAVD (difference from IOM of up to ± 20 mm) in 72% (34/47) and 70% (33/47) of women included, respectively, whilst MRI_Direct_ had good precision only in 47% (22/47) of women (Table 4).

**Table 4:** Proportion of lesion‐to‐anal‐verge distance measurements obtained using magnetic resonance imaging technique (MRI_Center_ or MRI_Direct_) or transvaginal sonography (TVS) according to their precision compared with intraoperative measurement (IOM), in 47 women with rectosigmoid endometriosis

Difference between measuring methods	MRI_Center_ *vs* IOM (*n* = 47)	MRI_Direct_ *vs* IOM (*n* = 47)	TVS *vs* IOM (*n* = 47)
± 20 mm (good precision)	34 (72)	22 (47)	33 (70)
> ± 20 to ± 30 mm (moderate precision)	8 (17)	8 (17)	7 (15)
> ± 30 to ± 50 mm (low precision)	2 (4)	12 (26)	5 (11)
> ± 50 mm (completely imprecise)	3 (6)	5 (11)	2 (4)

Data are given as *n* (%).

## DISCUSSION

To the best of our knowledge, this is the first study to compare TVS, based on the IDEA consensus, and MRI with IOM for estimating the location of a rectosigmoid endometriotic lesion. This study shows that the MRI_Center_ method is comparable to TVS when measuring LAVD. The comparison of MRI_Center_ and TVS with IOM shows that the methods are accurate, as reflected by the small mean difference between the methods. However, all methods were imprecise for measuring LAVD within the preset limit of ± 20 mm from IOM. MRI_Direct_ is less precise for lesions located further from the anal verge. There was a tendency for TVS and MRI_Direct_ to underestimate LAVD and for MRI_Center_ to overestimate LAVD.

Compared with IOM, LAVD measurements obtained by all methods were outside the preset clinically acceptable LoA of ± 20 mm. However, these limits may be too strict, as a previous study comparing MRI and rigid sigmoidoscopy among 99 rectal cancer patients found 95% LoA of −26 to 28 mm^22^. The authors did not mention the acceptable limits between the measuring methods. In contrast to this study, they included rectal cancer patients with tumor location < 150 mm from the anal verge. A clear anatomical definition or anal verge marker was not stated. To the best of our knowledge, there has been only one other study evaluating the accuracy of LAVD measurements for rectosigmoid endometriosis on TVS compared with IOM in 133 women, which found that TVS was accurate, i.e. no systematic differences in estimating LAVD compared with IOM^10^. However, this study and previous research demonstrate LoA outside the clinically preset acceptable difference of ± 20 mm between the measuring methods. This shows that there is a degree of uncertainty for both TVS and MRI when compared with IOM if the study is repeated in similar subjects under similar conditions. However, our results show good‐to‐moderate precision by both MRI_Center_ and TVS in up to 89% and 85%, respectively, and in 64% by MRI_Direct_ (Table 4). A study in rectal cancer patients^23^ found a fair correlation between rectoscopy and three different MRI measurements in estimating tumor height, reporting similar SD for all methods, in agreement with this study. Bowel preparation was used for MRI but not TVS, which reflects our clinical practice. However, MRI did not perform better than TVS in measuring LAVD.

One benefit of MRI examination is that high sigmoid endometriotic lesions (≥ 200–250 mm) are visible and easier to follow as the bowel gets more tortuous. DE lesions with this location cannot be visualized by TVS. The occurrence of lesions at this location does not have the same clinical significance, as lesions closer to the anal verge are more at risk of anastomotic leakage. Additionally, TVS has limitations in the assessment of DE on the pelvic side wall and especially assessment of ureters cranial to the uterine artery, which are not visible on TVS^24^, ^25^. Extrapelvic DE is also detectable on MRI^26^. MRI images can be stored and reinterpreted by other radiologists. This is also the case for TVS; however, it is a dynamic imaging tool, in which clinical history, examination and tenderness can guide the examination^27^. Nevertheless, both TVS and MRI_Center_ showed similar variability compared with IOM. Both imaging modalities depend on the experience of the examiner, although the learning curve for detecting rectosigmoid endometriosis is attainable^28^.

The main limitation of this study is the comparison of TVS and MRI measurements with IOM as the reference standard. There are several factors affecting the IOM method. First, when TVS and MRI measure LAVD, the DE lesion is usually attached to the uterus or other pelvic structures. IOM is performed after surgical detachment of the DE lesion. The bowel lesion is often mobilized, which may explain the variability of the results and the underestimation of LAVD by TVS. However, tumor height measurements in rectal cancer also vary when the tumor is fixed^14^. The use of IOM is another limitation because it is only an estimation of LAVD, but it is the closest method to a reference standard. Unlike rectal cancer, rectosigmoid endometriosis rarely infiltrates the bowel mucosa^29^. Thus, preoperative rectoscopy is not appropriate to use in women with DE. Conversely, MRI_Center_ with more than one caliper line follows the bowel lumen more than a rigid rectal probe or TVS probe, thus overestimating LAVD compared with IOM, whilst MRI_Direct_ underestimates LAVD, as one caliper line is used. Fibrosis and adhesions of DE lesions may alter bowel motility, which may affect the IOM measurements of the detached lesion^30^. In contrast, MRI allows still images of a fixed lesion, unaffected by bowel motility. Anesthesia may alter rectal length, affecting IOM^31^. The different positioning of the woman (supine position for MRI, lithotomy position for TVS and lithotomy–Trendelenburg position for IOM) may have had an effect on LAVD measurements. Moreover, unlike IOM, TVS and MRI methods use only an approximation of the anal verge. Inter‐ and intrarater variability was not assessed in this study, which is another limitation. Finally, this study was conducted in a tertiary referral center for endometriosis with a specialist radiologist and gynecologist, which may limit the generalizability of the results.

From a patient and surgical perspective, it is important to know the details of DE extent to improve counseling and treatment. We demonstrate that TVS and MRI are both useful diagnostic tools to examine systematically women with respect to LAVD. All the investigated measuring methods have limitations, as discussed above, but TVS is more accessible than MRI, which may avoid delays in diagnosis and optimize surgical treatment of women with rectosigmoid endometriosis. Additionally, LAVD should be recorded using the #Enzian classification^5^ for effective communication for clinical and research purposes.

In conclusion, TVS and MRI have similar performance in measuring LAVD when compared to IOM with a rectal probe as the reference standard. However, both methods have limitations, as discussed. Both methods represent an estimation of the LAVD. Detailed mapping of the full extent of DE is advisable when planning surgical strategy. TVS is more readily available and cheaper than MRI, which can help avoid delay in diagnosis and surgical treatment of rectosigmoid endometriosis, as well as improve counseling of women before surgery.

## Supporting information


**Figure S1** Bland–Altman plot showing differences in measurement of lesion‐to‐anal‐verge distance (LAVD) between magnetic resonance imaging (MRI)_Center_ method (a), transvaginal sonography (TVS) (b) and MRI_Direct_ method (c) compared with intraoperative measurement (IOM) plotted against the mean of measurements of each pair of methods, in 47 women with rectosigmoid endometriosis. Mean (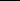) and limits of agreement (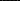) are displayed.

## Data Availability

The data that support the findings of this study are available on request from the corresponding author. The data are not publicly available due to privacy or ethical restrictions.
