# Supplementary figures and images for: Lesion‐to‐anal‐verge distance in rectosigmoid endometriosis on transvaginal sonography vs magnetic resonance imaging: prospective study
Source: Ultrasound Obstet Gynecol. 2023 Feb 1;61(2):243–50. doi: 10.1002/uog.26083 (PMC10107681; doi:10.1002/uog.26083)

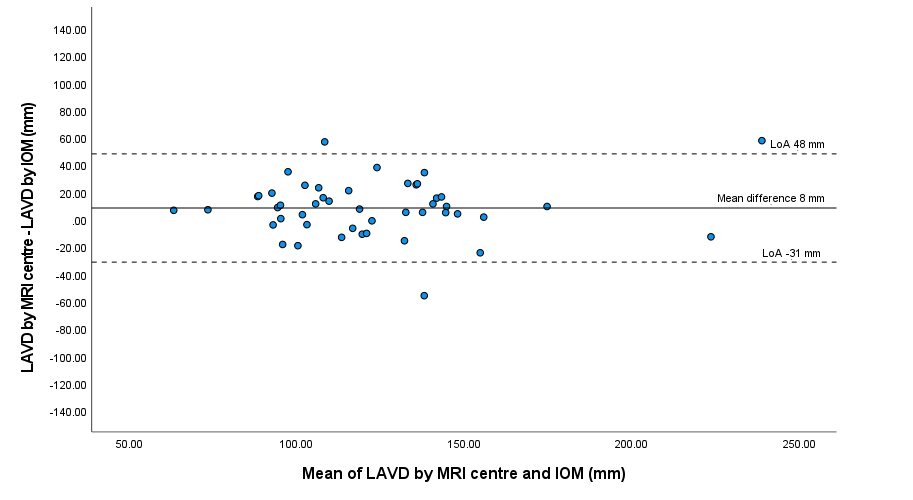

Supplement: Supplementary file 1 — Figure S1 Bland–Altman plot showing differences in measurement of lesion‐to‐anal‐verge distance (LAVD) between magnetic resonance imaging (MRI)Center method (a), transvaginal sonography (TVS) (b) and MRIDirect method (c) compared with intraoperative measurement (IOM) plotted against the mean of measurements of each pair of methods, in 47 women with rectosigmoid endometriosis. Mean () and limits of agreement () are displayed. [file UOG-61-243-s001.zip › UOG_26083_FigS1a_400_e.tif]

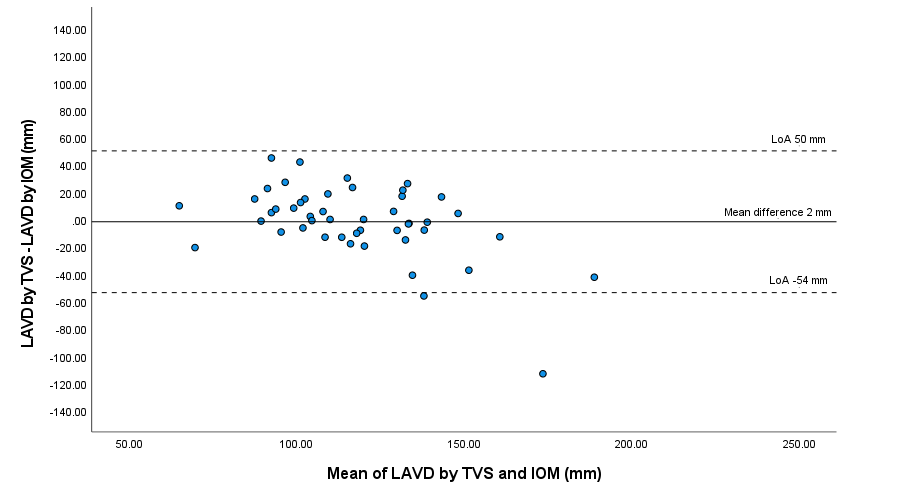

Supplement: Supplementary file 1 — Figure S1 Bland–Altman plot showing differences in measurement of lesion‐to‐anal‐verge distance (LAVD) between magnetic resonance imaging (MRI)Center method (a), transvaginal sonography (TVS) (b) and MRIDirect method (c) compared with intraoperative measurement (IOM) plotted against the mean of measurements of each pair of methods, in 47 women with rectosigmoid endometriosis. Mean () and limits of agreement () are displayed. [file UOG-61-243-s001.zip › UOG_26083_FigS1b_DG_e.tif]

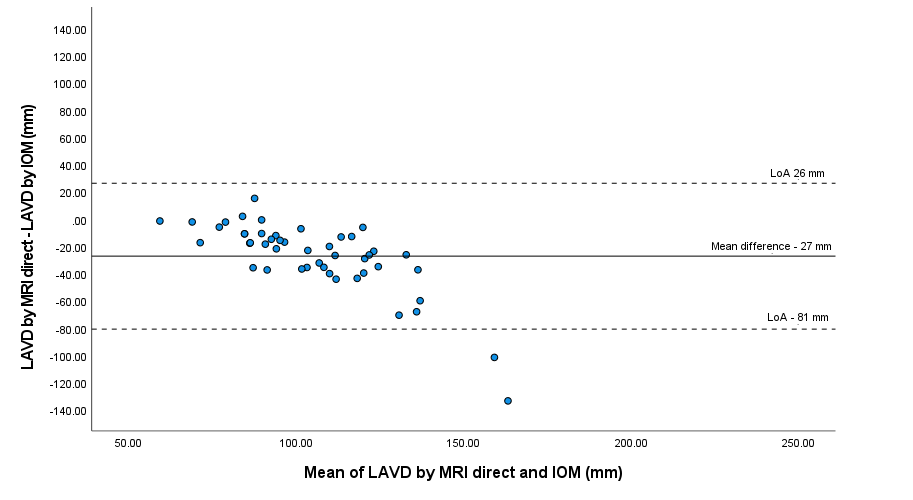

Supplement: Supplementary file 1 — Figure S1 Bland–Altman plot showing differences in measurement of lesion‐to‐anal‐verge distance (LAVD) between magnetic resonance imaging (MRI)Center method (a), transvaginal sonography (TVS) (b) and MRIDirect method (c) compared with intraoperative measurement (IOM) plotted against the mean of measurements of each pair of methods, in 47 women with rectosigmoid endometriosis. Mean () and limits of agreement () are displayed. [file UOG-61-243-s001.zip › UOG_26083_FigS1c_DG_e.tif]
